# Regulation of Viral Restriction by Post-Translational Modifications

**DOI:** 10.3390/v13112197

**Published:** 2021-11-01

**Authors:** Célia Chamontin, Guillaume Bossis, Sébastien Nisole, Nathalie J. Arhel, Ghizlane Maarifi

**Affiliations:** 1 Viral Trafficking, Restriction and Innate Signaling Team, Institut de Recherche en Infectiologie de Montpellier (IRIM), Université de Montpellier, CNRS, CEDEX 5, 34293 Montpellier, France; celia.chamontin@irim.cnrs.fr (C.C.); sebastien.nisole@irim.cnrs.fr (S.N.); nathalie.arhel@irim.cnrs.fr (N.J.A.); 2 The Ubiquitin Family in Hematologic Malignancies Team, Institut de Génétique Moléculaire de Montpellier (IGMM), Université de Montpellier, CNRS, CEDEX 5, 34293 Montpellier, France; guillaume.bossis@igmm.cnrs.fr

**Keywords:** restriction factors, intrinsic immunity, post-translational modifications, degradation

## Abstract

Intrinsic immunity is orchestrated by a wide range of host cellular proteins called restriction factors. They have the capacity to interfere with viral replication, and most of them are tightly regulated by interferons (IFNs). In addition, their regulation through post-translational modifications (PTMs) constitutes a major mechanism to shape their action positively or negatively. Following viral infection, restriction factor modification can be decisive. Palmitoylation of IFITM3, SUMOylation of MxA, SAMHD1 and TRIM5α or glycosylation of BST2 are some of those PTMs required for their antiviral activity. Nonetheless, for their benefit and by manipulating the PTMs machinery, viruses have evolved sophisticated mechanisms to counteract restriction factors. Indeed, many viral proteins evade restriction activity by inducing their ubiquitination and subsequent degradation. Studies on PTMs and their substrates are essential for the understanding of the antiviral defense mechanisms and provide a global vision of all possible regulations of the immune response at a given time and under specific infection conditions. Our aim was to provide an overview of current knowledge regarding the role of PTMs on restriction factors with an emphasis on their impact on viral replication.

## 1. Introduction

**Figure 1:**
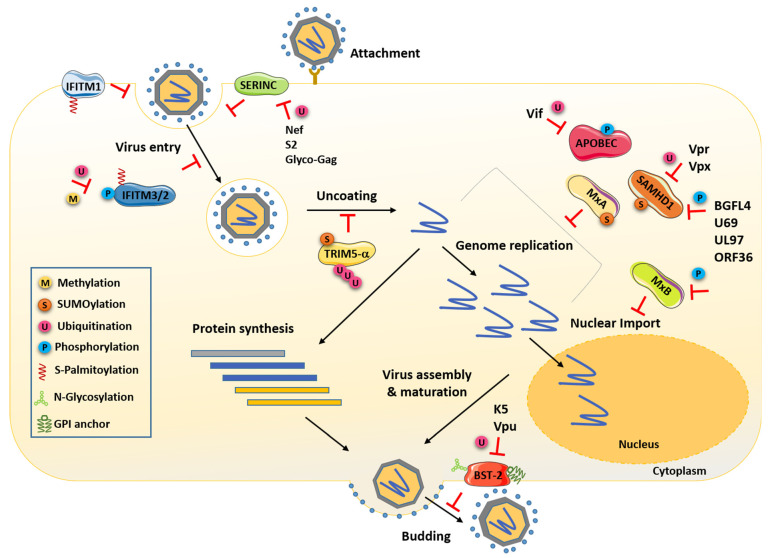
Cells express several cellular antiviral factors that interfere with almost every step of the viral replication cycle. Schematic representation that highlights the known PTMs of those factors required for their antiviral activity. The viral proteins and PTMs antagonizing these factors are also indicated. Nef, Vpr, Vpx, S2 and Glyco-Gag are retroviral proteins, and BGLF4, U69, UL97 and ORF36 are herpesvirus-encoded kinases.

Interferons (IFNs) constitute the first line of defense against pathogens and extracellular aggression. They orchestrate immune defenses through the induction of hundreds of genes named ISGs (IFN-stimulated genes). Moreover, there is another type of immunity, referred to as intrinsic immunity. It is mediated by antiviral proteins (defined as restriction factors) that display a potency to block specific steps of the viral replication cycle (Figure 1), acting as potent intrinsic barriers against infection [1]. While most antiviral factors are IFN-induced, some of these proteins are constitutively expressed [2].

Mx1 was the first antiviral factor discovered in 1962 for its capacity to interfere in an inbred mouse strain with influenza A virus (IAV) infection and in A2G mice with myxoviruses [3]. However, the term restriction factor was introduced in 1970 following the discovery of the retroviral restriction factor Fv1 that protects mice against infection by murine leukemia virus (MLV) [4]. Since, a variety of additional restriction factors have been described such as TRIM5α [5,6], APOBEC3G [7,8,9], SAMHD1 [10,11], Mx2/MxB [12,13,14], Tetherin/BST2 [15,16], SERINC3/5 [17,18] and IFITMs [19].

**Figure 2:**
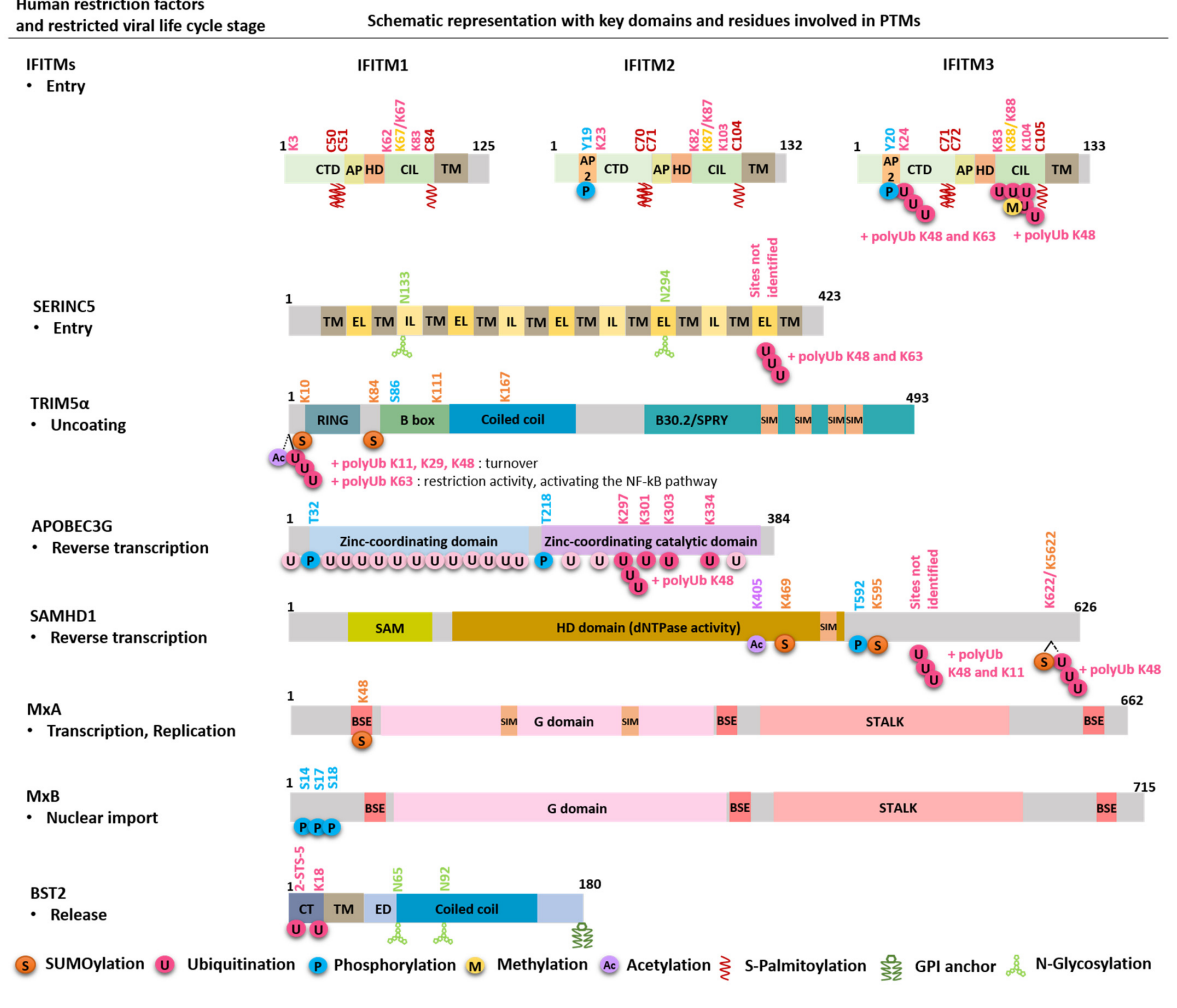
Schematic representation of human restriction factors, with key domains and residues involved in PTMs. For each restriction factor, the target viral step is indicated, and the modification residue and the corresponding PTM are marked with same colors. The 20 putative lysines (K) in APOBEC are K2, 40, 42, 52, 63, 76, 79, 99, 113, 141, 150, 163, 180, 249, 270, 297, 301, 303, 334 and 344. The four residues critical for Vif-induced APOBEC proteasomal degradation are indicated, although their importance remains controversial. CTD: cytoplasmic C-terminal domain; AP2: AP2 binding domain, AP: amphipathic helix; HD: hydrophobic domain; CIL: conserved intracellular loop; TM: transmembrane domain; IL: intracellular loop; EL: extracellular loop; SIM: SUMO-interacting motif; SAM: sterile alpha motif; HD domain: histidine-aspartic-containing domain; BSE: bundle-signaling element; G domain: GTPase domain; CT: cytoplasmic tail; ED: extracellular domain.

**Figure 3:**
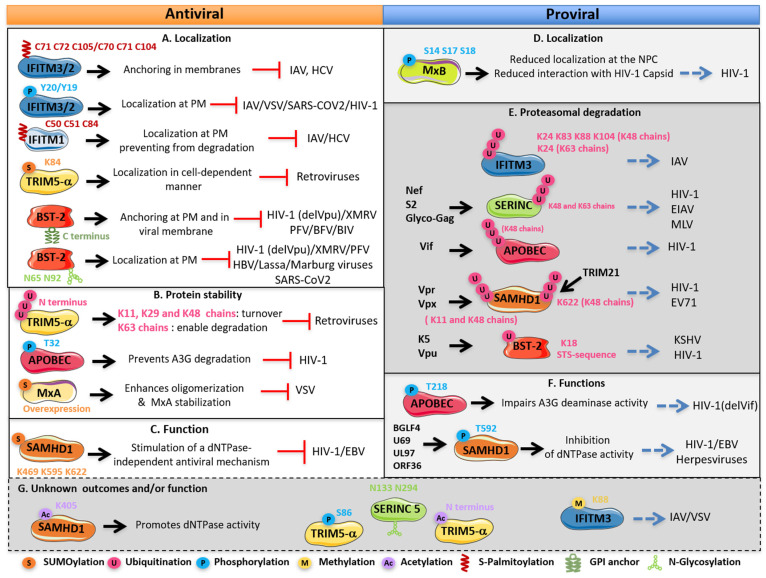
Regulation of human restriction factor activities and outcomes by PTMs. (**A**,**B**,**C**) Antiviral effects of the PTMs, (**D**,**E**,**F**) proviral effects of the PTMs on the restriction factors and (**G**) unknown outcomes and/or function of the PTMs on the restriction factors. Restriction factors with the modified residues are illustrated. For each factor, the mechanism involved, the outcome of modified factors, the effect on viruses and the viral proteins antagonizing these factors are indicated. PM: plasma membrane; NPC: nuclear pore complex.

As mentioned, the expression of most restriction factors is upregulated by type I IFNs, and this induction, therefore, constitutes the main mechanism of their regulation. However, more intricate levels of regulation have been brought to light. Indeed, post-translational modifications (PTMs) form a critical part of restriction factor regulation (Figure 1, Figure 2 and Figure 3). They can finely modulate their expression, conformation, localization, interactome, stability and therefore their functions and capacity to restrict viruses. Interestingly, these factors can be counteracted by viruses, often through hijacking PTMs for their own replication, thus forming another mechanism for their regulation.

In eukaryotic cells, proteins can undergo a wide variety of reversible and irreversible PTMs. Four major types of PTMs are documented in literature: (i) cleavage and proteolysis of proteins, (ii) the addition of proteins or polypeptides including ubiquitination and ubiquitin-like proteins (Ublps), (iii) the addition of complex molecules such as glycosylation and palmitoylation and finally (iv) chemical changes which include phosphorylation, methylation or acetylation [20,21]. The same protein can be affected by different modifications sequentially or in response to different cellular stimuli (virus infection, stress, cell cycle, etc.). In addition, PTMs can interact with each other, modify each other and/or modify the same target in a cooperative or competitive manner. They are involved in numerous biological and cellular processes including regulation of transcription, genome integrity, cell signaling, protein degradation, IFN pathway, host–virus interactions and innate immunity [22,23,24]. Phosphorylation, methylation, acetylation, ubiquitination, SUMOylation or even glycosylation are among the most studied PTMs and are also gaining importance in the context of antiviral factor regulation.

The last years of research on PTMs have revealed the potential of these modifications in innate immunity, and the regulation of host–pathogen interactions has received considerable research interest. Emerging evidence supports that PTMs form an interface between viruses, restriction factors and cellular defense mechanisms. Thus, identifying modifications of restriction factors has become a priority in understanding the mechanisms of innate immunity, antiviral defense and IFN response (Figure 1, Figure 2 and Figure 3).

This review summarizes the recent and well-characterized regulation mechanisms of restriction factors by PTMs, highlights the importance of these regulations at the interface of virus–cell defenses and gathers many examples illustrating this diversity of the consequences of PTMs.

## 2. Post-Translational Modifications (PTMs)

### 2.1. PTMs Based on the Addition of Polypeptides: Ubiquitination and SUMOylation

Ubiquitination consists in the covalent conjugation of ubiquitin, a highly conserved protein, to lysine (K) residues of target proteins. Its main documented function is to target its substrates to the main cellular degradation machinery, the proteasome [25]. This dynamic modification implicates three main enzymatic steps involving three types of enzymes: 2 E1 ubiquitin-activating enzymes, approximately 40 E2 ubiquitin-conjugating enzymes and around 700 E3 ubiquitin ligases. It can be reversed by around 100 different deubiquitinases (DUBs) in humans [26,27].

The most common types of ubiquitination are the modification by a single ubiquitin moiety (mono-ubiquitination) or poly-ubiquitination in which several ubiquitin proteins are added in a chain at the same position on the protein substrate. Poly-ubiquitination takes place by connecting new ubiquitin proteins to either a K or Methionine (M) residue of the previous ubiquitin molecule, thus forming a chain. Indeed, ubiquitin itself contains seven K residues (K6, K11, K27, K29, K33, K48 and K63) and the N-terminal M residue to which another ubiquitin can be conjugated. The most common types of poly-ubiquitination chains are K48, K29 (which normally tags proteins for proteasomal degradation), K63, K11, K6 and M1 which, together with mono-ubiquitination, are involved in cell trafficking, signaling pathways, lysosomal degradation, activation/inactivation of enzymatic activities, translation and DNA repair [28,29,30]. Nevertheless, in addition to ensuring the turnover of cellular protein and mentioned processes, ubiquitination constitutes a main regulatory mechanism allowing viruses to evade the action of restriction factors. SERINC5 [31,32], BST2 [33,34], APOBEC [35] and SAMHD1 [36] are some of those factors highly ubiquitinated and therefore targeted to their degradation by viral proteins or under conditions of infection (Figure 2 and Figure 3).

Thus, target proteins can be subjected to a variety of ubiquitin linkage types including mono-ubiquitination, modification by multiple single ubiquitin moieties (multiubiquitination), modification of non-canonical residues serine (Ser or S), cysteine (Cys or C) or threonine (Thr or T) and, finally, modification by polyubiquitin chains on K residue or N-terminal residue of target substrates [29,37], together illustrating the diversity and complexity of regulation of the ubiquitination signal.

SUMOylation is orchestrated by the small ubiquitin-like modifier (SUMO) proteins that belong to the ubiquitin-like (UBL) family. In humans, five paralogs of SUMO are described [38]. However, only SUMO1, 2 and 3 are well documented. These small proteins share an important structural identity with ubiquitin despite a low percentage of sequence identity [39]. Like ubiquitination, SUMOylation is an enzymatic reaction that also involves three types of enzymes: the E1-activating enzymes SAE1/SAE2, the E2-conjugating enzyme Ubc9 and one of the several E3 ligases (PIAS1, PIAS3, PIASxα, PIASxβ, PIASy, RanBP2, ZNF451, Pc2, etc.). SUMOylation is a reversible mechanism since SUMO can be deconjugated by the SENP proteins family, thus allowing the recycling of SUMO proteins [38,40]. SUMOylation consists of the covalent conjugation of SUMO on a consensus motif on a K residue, although alternative consensus sites have been identified [38]. SUMO2 and SUMO3 comprise a consensus sequence of SUMOylation at their N-terminal and can themselves be modified at lysine in position 11, K11. Thus, modification by SUMO2 and SUMO3 is characterized by their ability to form polySUMO2/3 chains, and they are often indicated as SUMO2/3 [38]. Recently, other lysines on SUMO2/3, K7, K21 and K33 have been reported to participate in chain formation [38]. Surprisingly, SUMO1, previously thought to act as a chain terminator, can also be modified on K7 by SUMO2/3 and can form polySUMO chains [38,41,42]. In contrast to other PTMs, SUMO can also interact non-covalently with target proteins bearing motifs called SIMs (SUMO-interacting motifs) via its SIG domain (SUMO-interacting groove) [38]. Both covalent and non-covalent interactions of target proteins with SUMO are key regulators for their activities.

SUMOylation modulates protein stability, interactions, subcellular localization and the activity of SUMOylated targets in a cell- or stimuli-dependent manner. SUMOylation leads to significant structural and conformational changes of the substrate by masking or conferring additional binding surfaces for protein interactions thereby modulating several cellular processes including signaling pathways, transcriptional regulation and protein stability [38,40]. In the last years, several studies revealed that SUMOylation also helps to regulate host immunity and appears, in many cases, to contribute to an antiviral state. Indeed, we and others have shown that various antiviral factors are SUMOylated (PML, PKR, MxA, TRIM5α, SAMHD1) [43,44,45,46,47,48,49,50] or non-covalently modified by SUMO (TRIM5α, Daxx) [51,52,53]. Their modifications finely modulate their restriction activities [24,54].

### 2.2. PTMs Based on the Addition of Functional Groups: Glycosylation and S-Palmitoylation

Glycosylation consists in the addition of sugar on proteins or lipids. In mammalian cells, glycosylation results in a wide variety of glycosidic linkages, catalyzed by different types of enzymes—glycosidases, glycosyltransferases and nucleotide sugar transporters, which can be divided into four major types: *N*-linked and *O*-linked glycosylation, *C*-linked mannosylation and glypiation [55]. The most prevalent is N-glycosylation, which consists in the attachment of a carbohydrate on one or more asparagine (N) residues on an acceptor site N-X-T/S. It takes place in the endoplasmic reticulum (ER) concomitantly with translation before processing along the Golgi pathway where they acquire their mature and complex form [55,56]. Proteins can also be subjected to another type of glycosylation, the GPI (glycosylphosphatidylinositol) anchor. This modification is catalyzed by a family of GPI protein transamidases and consists in the addition of a glycolipid on the hydrophobic C-terminal end of proteins [57]. Like other types of glycosylation, a GPI precursor is synthesized in the ER where it is directly attached to the protein. Modified proteins then traffic through the Golgi where GPI undergoes maturation. This review focuses on the role of two well-documented glycosylated factors, SERINC5 and BST2 [58,59] (Figure 2 and Figure 3).

S-Palmitoylation (S-acylation) mediates the covalent attachment of fatty acids, primarily palmitic acid composed of 16 carbons, to a cysteine residue via a thioester linkage [60]. This modification enhances the hydrophobicity of proteins and contributes to membrane attachment. Palmitoylation is catalyzed, in mammalian cells, by a family of 24 transmembrane proteins named DHHC palmitoyltransferases (PATs) all of which contain a conserved catalytic Zinc domain called DHHC catalytic domain (zDHHC). Each PAT has a specific subcellular location. However, most of them are resident in the Golgi. Due to this distribution, PATs control the association of palmitoylated proteins to the plasma membrane (PM) and other intracellular membranes. Inversely, the reaction is reversed by the acyl-protein thioesterases, which induce depalmitoylation of targeted proteins leading to their translocation into the cytosol. Thus, palmitoylation allows modulating the subcellular location, membrane trafficking and therefore function of the palmitoylated proteins. The best known palmitoylated restriction factors are IFTIMs. Their palmitoylation constitutes a key regulator for their cell trafficking and antiviral function (Figure 2 and Figure 3) [61,62,63].

### 2.3. Protein Chemical Changes: Phosphorylation, Acetylation and Methylation

Phosphorylation consists in the addition of one or more phosphate groups (PO_4_) to proteins. This reversible PTM is one of the most common and important PTMs and chemical protein changes that occur in animal cells. Indeed, more than two-thirds of the proteins encoded by the human genome have been shown or predicted to be phosphorylated (for human phosphorylated proteins, see websites http://www.phosphosite.org/ (accessed on 28 September 2021) and http://www.phosphonet.ca/ (accessed on 28 September 2021)). Serine (Ser), tyrosine (Tyr or Y) and threonine (Thr) are the amino acids that are subjected to phosphorylation. However, in eukaryotic cells, about 86.4% of phosphorylation events occur on Ser, while only 11.8% and 1.8% occur on Thr and Tyr residues, respectively [64,65]. In humans, the phosphorylated state of proteins is mainly determined by the activity of approximately 568 protein kinases and 156 phosphatases on their substrates [66]. Many kinases and phosphatases are themselves phosphorylated, thereby forming mutually dependent and hierarchically regulated signaling loops and cascades [67]. Similar to other PTMs, protein phosphorylation is involved in the regulation of a broad spectrum of cellular processes and signal transduction including antiviral response [66]. As shown in Figure 2, restriction factors IFITM3/2 [62,68,69,70], APOBEC [71,72,73] and SAMHD1 [74,75,76,77] can be subjected to phosphorylation. It is notable that their phosphorylation can modulate their activity positively or negatively depending on targets and viruses (Figure 2 and Figure 3).

Acetylation refers to the addition of an acetyl group (CH3CO) in a protein. This reaction is catalyzed by various acetyltransferases [78]. Protein acetylation normally occurs in two distinct forms, which constitute the cell-wide acetylome. In humans, the first one occurs for approximately 80% of proteins that are co-translationally acetylated at the nascent polypeptide chains [78]. Nevertheless, this type of modification named N-terminal (Nt) acetylation can also occur post-translationally, and reactions are catalyzed by Nt-acetyltransferases (NATs) [78]. The second requires K residues and was first characterized on histones. These enzymatic reactions involve histone acetyltransferases (HATs). In contrast to Nt-acetylation, which is considered irreversible, the acetylation status of a lysine is reversible and tightly regulated by histone deacetylases (HDACs) [78]. However, many non-histone proteins have been identified as the substrates of HATs and HDACs, which consequently were renamed lysine (K) acetyltransferases (KATs) and deacetylases (KDACs) [78]. Thus, the antagonistic actions of these enzymes, KATs and KDACs, dynamically control the acetylation state of several proteins, their stability and their interactome and serve as an important mechanism for the epigenetic regulation of gene expression and diverse cellular processes, such as chromatin remodeling, cell division, nuclear transport and cell metabolism [79]. In contrast to other PTMs, the role of acetylation in antiviral response is not well documented. Nevertheless, some reports have recently identified some restriction factors to be acetylated, including SAMHD1 [80] and TRIM5α [81,82] (Figure 2 and Figure 3). These pioneering studies point to the fact that acetylation may play a crucial role in antiviral defenses.

Methylation, similar to phosphorylation and acetylation, refers to the transfer of one-carbon methyl groups (CH3) to lysine or arginine residues of protein substrates [83]. This process is achieved by two types of enzymes called arginine methyltransferases (PRMTs) and lysine methyltransferases (PKMTs) [83]. In stark contrast to other modifications, the global turnover of lysine methylation is low, suggesting that this modification is stable and not reversible. However, several studies have described the existence of lysine demethylases suggesting that the methylation can be reversible under tightly regulated conditions [83]. Like acetylation, methylation has been widely studied first in histones, and unlike acetylated lysine residues on histones, which are generally associated with the activation of gene expression, histone methylation can lead to gene activation or repression based on the target residue [83]. Moreover, the most studied process is DNA and histone methylation contributing to epigenetic regulation [84] including, of note, viral DNA [85]. Indeed, several studies evidenced the role of DNA and histone methylation in virus epigenetic regulation and their association with innate immune evasion by human viruses including HIV [86], other RNA viruses and DNA viruses [87,88,89]. In contrast, only IFITMs are reported to be methylated, evidencing that methylation may play a crucial role in antiviral factor regulation (Figure 2 and Figure 3). Thus, more research is required to better explore its effects on other factors [90].

## 3. Antiviral Factors Are Highly Regulated by PTMs

### 3.1. IFITM Proteins

The IFITM (interferon-induced transmembrane) proteins are one of the first lines of defense against viruses by blocking the fusion of viral and host membranes and virus entry [19] (Figure 1). Their mechanisms of action are not yet fully understood. Nevertheless, work on IFITMs converges toward a model where these factors modify the properties of the host membrane and consequently impact viral entry [91,92].

Three members of the IFITM protein family, IFITM1, 2 and 3, are described for their antiviral activity, and a cooperative action of these three IFITMs is required for maximal antiviral activity [93]. These factors act against a broad spectrum of viruses, mostly enveloped, including IAV and West Nile virus (WNV) [94,95]. Over the past 10 years, IFITMs have also emerged as HIV-1 inhibitors, interfering with both early and late viral replication steps thanks to their ability to control cellular and viral membrane fusion [69,96]. Recently, it has been also reported that overexpression of IFITMs restricts β-coronavirus SARS-CoV-2 infection, supporting results previously obtained upon human coronavirus OC43 infection [70,97,98]. However, endogenous IFITMs, in particular IFITM2, seem to promote SARS-CoV-2 entry and production in human lung cells [99]. Although studies on the role of IFITMs in SARS-CoV infection present conflicting results and therefore deserve more investigation, IFITMs seem to display a key role in viral restriction depending on their level of expression, their localization, cell types and viruses [100].

IFITM1, 2 and 3 share a similar structure, with two hydrophobic domains, including an amphipathic helix and a transmembrane domain, separated by a conserved intracellular loop. Only IFITM1 differs with its shorter cytoplasmic N-terminal tail required for its localization at the plasma membrane (PM) [91,94]. Indeed, IFITM2 and 3 are essentially intracellular and localize in late endosomes and lysosomes while IFITM1 localizes more at the PM [61,68,94]. This differential localization of IFITMs and even stability are mainly under PTM control which strongly modulates their mechanism of action and impacts their antiviral potency. Indeed, IFITMs are highly modified. The most characterized are IFITM3 modifications. Depending on its palmitoylation, phosphorylation, ubiquitination or methylation, its location, stability and therefore activity are strongly regulated (Figure 3). Interestingly, these PTMs can occur at the same time or influence each other, increasing the possibility of IFITMs’ regulation and therefore their functions.

#### 3.1.1. S-Palmitoylation of IFITM Is Required for Its Localization and Antiviral Activity

IFITMs are part of the best-studied S-palmitoylated innate-immunity-associated proteins. Indeed, the three IFITMs undergo S-palmitoylation, although the most characterized is IFITM3, which can be S-palmitoylated at least on one of the three cysteines 71, 72 and 105 (Figure 2) [101,102]. This modification allows its membrane association in clusters in the ER [101], and loss of *S*-palmitoylation reduces the IFITM3 antiviral activity against IAV and Dengue virus [103,104]. The C72 residue is the main site of S-palmitoylation required for the antiviral activity of IFITM3 (Figure 1) [93,102]. While C105 is located close to the transmembrane domain, C71 and 72 are located nearby the amphipathic helix required to block virus–cell fusion. This modification may allow the anchoring of the amphipathic helix in the membrane for proper activity (Figure 2) [91]. Various zDHHC enzymes are necessary to promote S-palmitoylation of IFITM3. This modification is required for the effective inhibition of IAV infection. zDHHC20 has the most potent effect regarding the rate of S-palmitoylation and the antiviral response when co-expressed with IFITM3. IFITM3 and zDHHC20 colocalize in lysosome whereas the other zDHHCs localize in the Golgi, suggesting that, where S-palmitoylation occurs, it may affect the antiviral activity [104]. This highlights the importance of site-specific palmitoylation of IFITM3 in their antiviral function. In addition to IFITM3, IFITM1 S-palmitoylation is described to regulate its activity and prevent its proteasomal degradation. Indeed, a loss of IFITM1 palmitoylation in a murine model affects IFITM1 localization, stability and therefore its antiviral activity against IAV [105]. Likewise, the S-palmitoylation of all three IFITM proteins is essential for antiviral activity against Hepatitis C Virus (HCV) [62].

#### 3.1.2. Phosphorylation of IFITM3 Regulates Its Cell Trafficking and Promotes Its Antiviral Activity

IFITM3 and IFITM2 are also phosphorylated at Y20 and Y19, respectively, whereas IFITM1 is not [69]. This phosphorylation occurs in the N-terminal cytoplasmic tail by the membrane tyrosine kinase Fyn (Figure 2) [68]. This residue is located in a YXXφ sorting signal recognized by the adaptor protein 2 (AP2) complex that triggers IFITM3 and IFITM2 endocytosis [106].

Hence, phosphorylation regulates their trafficking within the cell: non-phosphorylated IFITM3 and IFITM2, which constitute a major part, are endocytosed from the PM and targeted to endosomes whereas phosphorylated IFITM3 and IFITM2 have their endocytosis signal masked and accumulate at the PM [68,107]. However, mutation of the tyrosine residues Y19 and Y20 to a phenylalanine induces an IFITM1-like phenotype and relocalizes IFITIM3 and IFITM2 to the cell surface [69]. Indeed, IFITM1 does not have the AP-2 motif and already exhibits a primary localization at PM and early endosomes [94,108]. The relocation of IFITM3 and IFITM2 following their phosphorylation is a dynamic and reversible process that certainly affects their activity upon a variety of viruses. However, while mutation of the tyrosine residue abrogates IFITM3 and IFITM2 activity against IAV, the vesicular stomatitis virus (VSV), HIV-1 and SARS-CoV-2 [68,70,106,107], this residue is dispensable for IFITM3 and IFITM2 anti-HCV activity (Figure 3A) [62].

#### 3.1.3. Ubiquitination of IFITM3 Promotes Its Degradation and Reduces Its Antiviral Activity

In addition to being S-palmitoylated and phosphorylated, IFITM3 holds four cytoplasmic lysines—K24, K83, K88 and K104—that can be all ubiquitinated. However, the prevalent one is K24 (Figure 2) [61]. These residues are conserved in IFITM1 and 2 but have not been investigated [61]. While IFITM3 phosphorylation and palmitoylation promote its antiviral activity, IFITM3 ubiquitination targets it for degradation and consequently limits its antiviral function (Figure 3E). The modification of IFITM3 through ubiquitination is mediated by the E3 ubiquitin ligase NEDD4 that binds to IFITM3 on a 17-PPxY-20 motif (where P is a proline, x is any amino acid, and Y is a tyrosine) on the N-terminus and initiates polyubiquitination via K48 or K63 linkages. K48 polyubiquitin chains are the most prevalent and target IFITM3 to degradation, thus interfering with the antiviral activity notably against IAV, while depleting NEDD4 restores IFITIM3 activity [61,109]. Interestingly, NEDD4 E3 ligase activity is inhibited by ISG15 [110]. Hence, upon IAV infection, the production of IFN, by inducing IFITM3 expression and inhibiting its ubiquitination—due to the concomitant induction of ISG15—promotes a positive regulation of IFITM3 expression and favors antiviral activity [19,109]. Interestingly, it is important to note that the ubiquitination of IFTIMs can be regulated by their phosphorylation. Indeed, while ubiquitination and S-palmitoylation are independent mechanisms and can occur at the same time [61], the phosphorylation of IFITM3 on Y20, which is included in the ubiquitination motif, walls out its ubiquitination and prevents its degradation [107]. This shows again how IFITM3 phosphorylation, by preventing its degradation in addition to its cell-trafficking regulation, promotes its antiviral activity.

#### 3.1.4. Methylation of IFITM Impairs Its Antiviral Effect

Besides the aforementioned modifications, IFITM3 can also be monomethylated on K88 which leads to a negative regulation of its antiviral activity [90] (Figure 2). Like IFITM3, IFITM1 and 2 are monomethylated by the PKMT Set7. However, only IFITM3 methylation has been characterized. IFITM3 methylation can be reversed by the demethyltransferase LSD1 enhancing its antiviral activity [111]. Thus, LSD1 plays a key role in IFITM3 activities during infection. Indeed, it has been reported that Zika virus infection triggers increased LSD1 expression and inversely decreased SET7 expression resulting in a reduction of methylated IFITM3. Furthermore, IFITM3 and LSD1 interactions are enhanced under IFN-α treatment promoting again its antiviral activity [111]. Inversely, VSV and IAV infections promote Set7 and IFITM3 interaction limiting the interaction with LSD1, which results in more methylated IFITM3. Despite the fact that IFITM3 methylation dramatically reduces its antiviral activity and capacity to restrict VSV and IAV (Figure 3G) [90,111], it does not affect its expression or even localization [90,111]. How methylation inactivates IFITM3 remains to be elucidated. Nevertheless, studies of IFITM3 regulation through its methylation reveal that, while IFN-α allows the activation of IFITM3 by inducing their demethylation, viruses such as VSV and IAV have evolved to counteract its action by interfering with this process, emphasizing the important role that modifications can play in host–pathogen interactions.

### 3.2. Ubiquitination of SERINC Proteins Lead to Its Degradation upon Infection

Serine incorporators (SERINC) 3 and 5 have been identified as restriction factors against retroviruses [17,18,112] and against hepatitis B virus (HBV) [113]. Recently, the antiviral action of SERINC4 has also been established against HIV-1 [114]. These factors belong to a family of five proteins, TMS–TDE family, characterized by 10 to 11 transmembrane domains (Figure 2) [115]. Importantly, of all the members in the SERINC family, only the SERINC3, 4 and 5 are restriction factors and have the ability to inhibit viral infection by blocking viral fusion (Figure 1). Indeed, SERINC3 and 5 are highly expressed at the PM of human peripheral blood mononuclear cells (PBMCs), where they incorporate into virions and inhibit the fusion of the virus with new target cells [17,116]. SERINC5 protein has five isoforms generated by alternative splicing. These differ at the terminal carbon end and in the transmembrane domains [117]. However, only the SERINC5-001 isoform is expressed at the PM with greater quantities compared to other isoforms, and it is the only isoform known to be involved in HIV restriction [18]. In contrast to retroviruses, the mechanism of inhibition of HBV seems to be different. Indeed, SERINC5 interferes with the glycosylation of HBV-envelope protein, inhibiting in this way the complete production of HBV virions [113].

During the evolutionary arms race, several viruses have developed strategies to counteract cellular factors by manipulating antiviral factor’s PTMs. Ubiquitination of SERINC proteins seems to be among these strategies. Indeed, SERINC3, 4 and 5 are counteracted by the accessory protein Nef (negative regulatory factor) encoded by HIV-1 [17,18,114], by the glycosylated Gag (glycoGag) of MLV [31] and by the small accessory protein S2 of equine infectious anemia virus (EIAV) [118]. These viral proteins all prevent SERINC5 incorporation into viral particles by inducing its ubiquitination and therefore downregulating their cell surface expression. For SERINC5, K48 and K63 polyubiquitination linkages are known to be a prerequisite for its lysosomal degradation (Figure 2 and Figure 3E) [31,32,119]. Viral proteins induce its endocytosis via clathrin-mediated trafficking by recruiting AP2, which will target the ubiquitinated SERINC5 to degradation [18,31,32,118,119]. However, the mechanisms of how viral proteins lead to SERINC protein ubiquitination are still unclear, and the ubiquitinated sites remain to be identified. Except for the ubiquitination of SERINC5, very little is known about SERINC3 and 4 regulations and even less about the regulation of all three by other PTMs. Indeed, only a recent study identified the residues N294 and N133 as glycosylated sites on SERINC5 (Figure 2 and Figure 3B) [58]. However, even if N294 glycosylation seems to be important for its stability, a loss of glycosylation neither affects SERINC5 activity against HIV-1 nor HBV [58,113]. Future research may provide us with more answers regarding the regulation of these proteins by other PTMs.

### 3.3. TRIM5α

Rhesus monkey (rh) and human (hu) TRIM5α (tripartite motif protein 5 α) are restriction factors that mediate species-specific anti-retroviral restriction [120]. rhTrim5α was identified to block HIV-1, EIAV and FIV, while huTrim5α counteracts N-MLV infection and more modestly FIV [5,121,122,123]. TRIM5α belongs to the TRIM protein family. These proteins share a common structure, with three conserved domains: a RING (really interesting new gene) domain usually associated with E3 ubiquitin ligase activity, one or two B-Box motifs and a coiled-coil region allowing their oligomerization. The C-terminus of huTrim5α and rhTrim5α possesses one B30.2 (also called PRYSPRY) domain (Figure 2). The latter recognizes and binds to the viral capsid and defines the restriction specificity [123,124]. Then the protein auto-oligomerizes and forms a hexagonal lattice around the capsid and triggers premature uncoating, thus aborting viral RNA reverse transcription (Figure 1) [125,126].

#### 3.3.1. TRIM5α Exerts Its Antiviral Activity through Auto-Polyubiquitination

The RING domain of TRIM5α exerts E3 ubiquitin ligase activity when assembled as a dimer [127]. First evidence showed that it has the ability to ubiquitinate itself in a mono- and poly-ubiquitinated manner [128].

On one hand, these ubiquitinations are branching via K11, K29 and K48 onto TRIM5α to trigger the rapid turnover of the protein (Figure 2 and Figure 3B) [82]. One possibility is that it could be achieved by the ubiquitin fusion degradation (UFD) machinery that targets N-terminal ubiquitin proteins for proteasomal degradation [82]. This probably allows maintaining a basal level of TRIM5α in the cell.

On the other hand, the restriction activity of TRIM5α is mediated via the K63 linkage type (Figure 2). The bundle of three contiguous RING domains mediates the elongation of K63–polyubiquitin chains with heterodimeric E2 enzymes Ube2N/Ube2V2 on the N-terminus [81,82]. While TRIM5α primary localizes diffusely in cytoplasm, ubiquitinated TRIM5α clusters in cytoplasmic bodies along with proteasome subunits, and this association can also occur in the context of viral infection [129,130]. Indeed, K63-linked ubiquitination enables proteasomal recruitment leading to TRIM5α degradation and virus disassembly [82]. It is known that premature uncoating disrupts viral DNA synthesis resulting in reverse transcription inhibition [131]. Thus, ubiquitin ligase activity of TRIM5α allows the inhibition of reverse transcription [81,130,132]. Alternatively, TRIM5α has also a key role in the activation of innate immune response through its ubiquitin ligase activity [6,130]. TRIM5α can act as a pattern recognition receptor (PRR), which recognizes the retroviral capsid as a pathogen-associated molecular pattern (PAMP). The resulting anchored K63–polyubiquitin chains on TRIM5α lead to the activation of the NF-κB pathway promoting antiviral activities [82]. Through their investigation to identify the sites of ubiquitination on Trim5α, Fletcher et al. brought to light N α-terminal acetylation of TRIM5α by mass spectrometry (Figure 2) [81,82]. Their studies showed that TRIM5α is expressed under both forms, acetylated and non-acetylated N-termini in cells. Interestingly, N-terminal ubiquitination can only take place on non-acetylated TRIM5α. The remaining questions are about the potential role of N-acetylation in TRIM5α regulation and the impact on its ubiquitination and consequently on its viral restriction (Figure 3G).

#### 3.3.2. SUMOylation of TRIM5α Regulates Its Antiviral Activity and Affects Its Subcellular Localization in a Cell-Dependent Manner

TRIM5α contains four putative SUMOylation consensus sequences and four putative SIM domains (Figure 2) [43,51,133] suggesting that SUMO proteins are crucial regulators of TRIM5α. Indeed, one consensus site was first identified as the major site of SUMOylation upstream of the RING domain at K10 residue. However, its mutation had little or no influence on TRIM5α restriction activity (Figure 2) [46,51,52]. Instead, this site seems to be involved in the activation of the TRIM5α-dependent innate immune response. K10 SUMOylation modulates the E3 ubiquitin ligase activities of TRIM5α promoting its K63 polyubiquitination and therefore activating the NK-κB pathway [134]. Several years later, our laboratory identified residues K84 and K85 as the main SUMOylated sites in cells of huTRIM5α and rhTRIM5α, respectively (Figure 2 and Figure 3) [43]. Their SUMOylation, which involves the SUMO-E3 ligase activity of RanBP2, is required for TRIM5α restriction activity. Interestingly, these lysines are part of a phosphorylated SUMOylation (pSUM) motif. Indeed, the serine at position 86 (S86) is predicted to be phosphorylated, suggesting that phosphorylation of TRIM5α may be a key regulator of its SUMOylation, influencing its antiviral activity in a cooperative or competitive manner (Figure 2 and Figure 3G). This potential interplay between phosphorylation and SUMOylation is a new promising lead to investigate. In addition, SUMOylation by RanBP2 has also been reported to modify TRIM5α localization [43,47]. Indeed, in dendritic cells, where TRIM5α is inactive, SUMO induces TRIM5α sequestration in the nucleus, where it cannot interfere with the viral capsid. In this case, the TRIM5α SUMO-dependent localization favors a good IFN response by the sensing of the retroviral DNA by the cytosolic sensor cGAS since reverse transcription is not disrupted [47]. As SUMOylation is reversible, this work describes an alternative mode of regulation of TRIM5α activity—SUMO-dependent—which allows TRIM5α to react depending on the cell type and the infection.

### 3.4. APOBEC3s Proteins

APOBEC3 proteins (apolipoprotein B mRNA editing enzyme, catalytic polypeptide-like) are a family of cytidine deaminases including seven members in humans (APOBEC3 A to H). They contain either one or two conserved zinc-coordinating domains, one of them being catalytically active (Figure 2) [135]. These factors target the reverse transcription step of the viral cycle. Thus, they harbor antiviral activity against endogenous retroelements, retroviruses and Hepatitis B virus (HBV), which replicate their DNA genomes by reverse transcription of an RNA intermediate [135]. Among this family of enzymes, APOBEC3G (A3G) was the first restriction factor, with a potent antiviral function against HIV-1, identified in 2002 [135]. A3G is stably expressed in lymphoid cells which are the main target of HIV-1 infection [7,136]. These deaminases are encapsidated into assembling viral particles and induce G to A hypermutation in newly synthesized retroviral DNA [8,9,137]. Thus, the viral reverse transcription is defective and hinders the productive infection (Figure 1). Like other restriction factors, APOBEC3 is regulated through PTMs. The most studied are ubiquitination and phosphorylation.

#### 3.4.1. Ubiquitination of APOBEC3 Is Useful for Viruses

Most lentiviruses express the accessory protein Vif that counteracts APOBEC3 antiviral activity through its ubiquitination (Figure 1) [35,136]. This mechanism has been extensively studied over the last twenty years and has been well reviewed [54,138]. Briefly, in order to prevent its encapsidation into viral particles, Vif binds to A3G on its N-terminal side and induces its polyubiquitination and subsequent proteasomal degradation. For this, Vif recruits an E3 ubiquitin ligase complex and the cofactor core-binding factor subunit beta (CBF-β) [139,140]. CBF-β is a transcriptional cofactor that is also known to regulate A3G transcription in CD4^+^ T cells [141]. Hence, through the hijack of CBF-β, Vif mediates A3G degradation on one hand and inhibits A3G transcription on the other hand. The complex recruited by Vif catalyzes the formation of K48-linked polyubiquitin chains on A3G which can occur through twenty putative K residues located all along A3G (Figure 2) [140,142]. However, the residues critical for Vif-induced APOBEC proteasomal degradation remain controversial (Figure 2) [142,143,144]. Indeed, although A3G mutants harboring only lysines available on the N-terminal side are still highly ubiquitinated, they are protected from degradation, and their antiviral activity is still functional [142]. Thus, the position of the ubiquitinated lysines on A3G modulates its stability and influences its outcome and activity upon infection.

Interestingly, the ubiquitin-specific protease USP49 was identified to positively regulate the expression of A3G through a screen on a deubiquitination enzyme (DUB)—siRNA library in HEK293T [145]. USP49 has the capacity to catalyze the K48-linked deubiquitination of A3G and increases its stability. Thus, USP49 counteracts the Vif effect and strengthens A3G against HIV-1. Of interest, the investigation of the expression of USP49 in CD4^+^ T cells of HIV-1 sero-positive patients reveals, in newly diagnosed individuals, a positive correlation between the expression of USP49 and A3G. In latently infected patients receiving anti-retroviral therapy, USP49 expression was correlated with G to A mutations in viral proteins and with less intact provirus. This suggests that USP49 could promote the formation of defective proviruses and in this way contains the viral reservoir.

APOBEC3 is also known to be subject to several polymorphisms impacting its antiviral activity [146]. This polymorphism can impact its ubiquitination. APOBEC3H (A3H) is very polymorphic, and there are four major variants in humans, haplotype II being the most active. Polymorphisms are often a sign of adaptation of the restriction factors toward pathogens. In the case of A3H and Vif proteins, there is indeed a close coevolution [147]. A recent study investigated the presence of polymorphisms in A3G from Chinese rhesus macaques and their effect on infection by the virus HIV-2 [148]. The authors identified a variant of A3G, A3G L71R, which protects A3G from HIV-2 Vif-mediated degradation through a change of its conformation. Inversely, a recent study showed that haplotypes I, III and IV in A3H undergo stronger ubiquitination regardless of the Vif effect, while haplotype II is more preserved from this modification [146]. However, inhibiting ubiquitination through K mutagenesis does not restore an effective antiviral function of haplotypes III and IV. Thus, the polymorphisms affect stability and activity independently. These results suggest that the regulation of the turnover of the proteins differs according to the variant expressed. The emergence of this defective polymorphism despite the efficiency of this restriction factor remains to be understood. To conclude, ubiquitination greatly influences APOBEC3, and a tight evolutionary struggle takes place between APOBEC and Vif to maintain its antiviral ability and defeat the lentiviral counteraction.

#### 3.4.2. Phosphorylation of APOBEC3: Divergent Actions Affecting Its Antiviral Activity

A3G is subjected to phosphorylation on T32 and T218 residues located upstream of each cytidine-deaminase domain (CDD). Its phosphorylation is regulated by the kinase PKA (Figure 2) [71,72]. However, depending on the residue, phosphorylation leads to distinct effects on its activity (Figure 3). Indeed, the first residue T32 has been characterized to confer robustness to A3G toward HIV-1 [71]. Phosphorylation on this residue interferes with the binding of the Vif protein, thus reducing its ubiquitination and consequently its degradation (Figure 3B). Importantly, kinase PKA, as A3G, is incorporated in viral particles [149], where its action could take place. Inversely, phosphorylation on T218 was described to impair A3G deaminase activity and further interfere with its antiviral activity (Figure 3F) [72]. Indeed, phospho-mimetic A3G-T218E had a diminished antiviral activity toward Vif-deficient HIV-1 even if its DNA binding ability was not affected. This regulation would allow to switch off the action of A3G in normal conditions, protecting mammalian genomic DNA from mutations, and conversely, in an infection context, phosphorylation would allow a fast activation [72].

As A3G, APOBEC3B (A3B) is phosphorylated by the PKA at Thr214 located in the CDD [73]. However, for A3B, phosphorylation affects the binding of ssDNA to the catalytic core, rendering its deaminase activity ineffective. In contrast to A3G, the anti-retroviral activity of A3B was only partially reduced by phosphorylation.

### 3.5. SAMHD1

SAMHD1 (sterile α motif (SAM) domain and histidine-aspartate HD domain-containing protein 1) is a triphosphohydrolase (dNTPase) (Figure 2) [150,151] and a major restriction factor identified for its capacity to restrict HIV-1 in dendritic and myeloid cells. By hydrolyzing cellular dNTPs, SAMHD1 interferes with HIV-1 reverse transcription and therefore inhibits HIV-1 infection (Figure 1) [10,11,152,153]. Since then, a broad-spectrum activity against RNA and DNA viruses has been demonstrated [150,151]. Moreover, SAMHD1 is described to play several cellular functions as its dNTPase activity is crucial to regulate key cellular mechanisms. Indeed, SAMHD1 is involved in cell cycle regulation and proliferation, replication fork progression, apoptosis, innate immunity and DNA damage response [150,151]. SAMHD1 is highly modified by PTMs, and the effect of these PTMs on SAMHD1 activity against HIV-1 is mainly investigated.

#### 3.5.1. Inactivation of SAMHD1 through Phosphorylation

SAMHD1 is phosphorylated at residue T592 by the complex composed of the cyclin A2 with the cyclin-dependent kinases 1 or 2 (CDK1/2) (Figure 2). This modification, which occurs in proliferating cells, inhibits the capacity of SAMHD1 to restrict HIV-1 while the unphosphorylated form is active in non-cycling cells [74,76,77]. SAMHD1 phosphorylation also negatively regulates its activity in a mouse model [154].

The regulation of SAMHD1 through its phosphorylation is well documented. Indeed, the primary role of SAMHD1 is to control the level of dNTP during the cell cycle. During the S phase, the cyclin-A2–CDK2 complex initiates SAMHD1 phosphorylation, which is maintained during the G2 phase by the cyclin-A2–CDK1 complex. Therefore, inactivating SAMHD1 during the S phase promotes a high level of dNTPs to allow cell DNA synthesis before mitosis. Conversely, in the G1 phase, SAMHD1 is unphosphorylated thanks to the phosphatase PP2A-B55α and hence active, coincident with a decrease in dNTP level [155,156]. In cycling cells, SAMHD1 dephosphorylation occurs during mitotic exit and might allow reducing HIV-1 reverse transcription during the G1 phase. Type I, II and III IFNs promote the downregulation of CDK1 expression and enhance PP2A-B55α expression, leading to dephosphorylation of SAMHD1 and thus its activation [156,157]. However, how phosphorylation inactivates SAMHD1 is still unclear. Indeed, diverging results were obtained concerning the effect of phosphorylation on its dNTPase capacity [155,158]. Furthermore, phosphorylated SAMHD1 preserves its stability, localization and oligomerization ability [76,155].

Nevertheless, viruses evolved to promote SAMHD1 phosphorylation to shut its action off. BGLF4, a kinase encoded by Epstein–Barr virus (EBV) phosphorylates SAMHD1 on T592, which inhibits its dNTPase activity [159]. Moreover, other kinases from beta- and gamma-herpesviruses also trigger the same phosphorylation: human herpesvirus 6/7 (HHV-6/7) U69, human cytomegalovirus (HCMV) UL97 and Kaposi sarcoma-associated herpesvirus (KSHV) ORF36, suggesting a shared mechanism of action of these different viruses (Figure 1 and Figure 3F) [159]. This highlights that targeting the phosphorylation of SAMHD1 may be an effective way to control various infections. Indeed, tyrosine kinase inhibitors, such as dasatinib, which block SAMHD1 phosphorylation and preserve its activity with a potent effect against HIV-1 reverse transcription, are one of the promising ways to reduce the HIV-1 reservoir [157,160].

#### 3.5.2. A Potential Role of Acetylation and SUMOylation in the Regulation of SAMHD1 Functions

Recently, SAMHD1 was reported to be SUMOylated and also acetylated. However, while SUMOylation of SAMHD1 clearly impacts its activity, there is not yet enough insight to conclude on the potential effects of SAMHD1 acetylation on its activity. SAMHD1 is acetylated on the K405 residue by an acetyltransferase arrest-defective protein 1 (ARD1) (Figure 2) [80]. The rate of SAMHD1 acetylation is highest during the G1 phase and promotes enhanced dNTPase activity. Acetylation of SAMHD1 is also reported to promote the G1/S transition and, consequently, cancer cell proliferation. However, the implication of this PTM on the antiviral activity of SAMHD1 has not been yet investigated (Figure 3G). It would be interesting to decipher the impact of this modification on the inhibition of the viral reverse transcription by SAMHD1.

In contrast to acetylation, SUMOylation of SAMHD1 seems to regulate its antiviral activity. Indeed, SAMHD1 was recently described as SUMOylated by the E3 SUMO ligase protein inhibitor of activated STAT 1 (PIAS1) on three lysine residues (K469, K595 and K622) (Figure 2) [50,161]. SUMOylation of SAMHD1 is required for both EBV and HIV-1 restriction (Figure 1 and Figure 3C) [50,161]. The antiviral activity of SAMHD1, against HIV-1, relies on the mono-SUMOylation of the K595 residue located in the CDK-targeted motif driving T592 phosphorylation (592-TPQK-595). SUMOylation of SAMHD1 and its HIV-1 inhibition are also dependent on a SIM motif located upstream of the T592 motif (Figure 2) [50]. Strikingly, this modification can occur simultaneously with phosphorylation despite their opposite effect on the antiviral activity. In non-cycling cells, Martinat and coworkers revealed a model where SAMHD1 has to be both unphosphorylated and SUMOylated on K595 to be antivirally active, although its modification by SUMO2 does not influence its dNTPase activity [50]. Thus, SUMOylation of SAMHD1 stimulates a dNTPase-independent antiviral mechanism. Nevertheless, additional work is needed to unravel the precise role and mechanisms of action of these PTMs on SAMHD1 outcomes and activities.

#### 3.5.3. Lentiviral Antagonism by Ubiquitination of SAMHD1

In the case of SAMHD1, ubiquitination is the main PTM used by viruses to escape antiviral activity and to promote their own replication [162]. Indeed, primate lentiviral lineages encode either the viral protein x (Vpx) or the viral protein R (Vpr), which in some strains, share the same ability to use the ubiquitin–proteasome system to induce SAMHD1 degradation (Figure 1) [10,36,163]. Vpx and Vpr are known to redirect the CRL4 E3 ubiquitin ligase to target SAMHD1 [36]. SAMHD1 is then polyubiquitinated through K48 and K11 linkages and targeted for proteasomal degradation (Figure 2 and Figure 3E) [164]. However, the CRL4 E3 ubiquitin ligase can itself be regulated through its ubiquitination by NEDD8 leading to its activation [165]. Consequently, blocking NEDD8 activity through the use of the MLN4924 drug interferes with the capacity of Vpx to induce SAMHD1 ubiquitination and degradation [166].

Moreover, another mechanism of regulation of SAMHD1 involving its ubiquitination has been reported. Indeed, the E3 ubiquitin ligase TRIM21 was lastly identified as a new regulator of SAMHD1 degradation [167], and overexpression of TRIM21 was correlated with the loss of both SAMHD1 expression and viral restriction against enterovirus 71 (EV71) and HIV-1. Upon infection, type I IFN production leads to the upregulation of TRIM21 expression that directly binds to SAMHD1, promotes its polyubiquitination at K622 via K48 chains and ultimately its degradation (Figure 2 and Figure 3E). This work reveals a new mechanism allowing viruses to hijack the IFN response by taking advantage of restriction factor modification by PTMs and highlights again the fact that ubiquitination of cellular or viral components is a major regulator of interactions between viruses and innate immunity effectors. Importantly, SAMHD1 ubiquitination competes with its SUMOylation, suggesting that SUMOylation, by blocking SAMHD1 ubiquitination and degradation, could enhance its antiviral activity (Figure 2) [161]. Once again, this emphasizes the importance of the interactions between PTMs and restriction factor functions.

### 3.6. MxA and MxB

Myxovirus resistance proteins are found in nearly all vertebrates, and most mammals encode for two Mx proteins named MxA and MxB in humans [168]. Mx proteins are IFN-induced GTPases composed of an N-terminal G (GTPase) domain which binds and hydrolyzes GTP, a stalk domain made of a middle domain, a C-terminal GTPase effector domain and three bundle-signaling elements (BSEs) that link these two domains for proper spatial conformation (Figure 2). To have functional GTP hydrolysis activity and effective antiviral activity, MxA has to assemble in oligomers via the stalk region [168]. MxA has been described to have a cytoplasmic localization where it restricts a wide range of viruses. Its mechanism of action differs substantially according to the virus and the species. It usually takes place in the early stages of the viral infection. For example, MxA targets VSV and IAV nucleocapsid to inhibit transcription, while it traps the N protein of La Crosse virus (LACV) to block viral replication (Figure 1) [168]. Concerning MxB, its restriction ability has been principally studied against HIV-1. MxB is located on nuclear pores where it impairs the import of HIV-1 pre-integration complex into the nucleus (Figure 1) [12,13]. Little is known about MxA and MxB modifications. For now, SUMOylation has been described to regulate MxA, while phosphorylation has been recently pointed out to regulate MxB.

#### 3.6.1. SUMOylation Regulates MxA Antiviral Activity

Several reports demonstrate that mouse and human MxA proteins interact with different components of the SUMO machinery and that SUMOylation regulates its antiviral activity. Indeed, MxA interacts with the EIL loop of SUMO1 in a SIM-independent manner via its stalk (CID-GED) domain, with the subunit 2 of the SUMO1-activating enzyme (SAE2) and with Ubc9 via the GTPase domain [45]. The oligomerization of MxA seems to be important for these interactions [45]. MxA is SUMOylated at the K48 residue located in the first BSE (Figure 2) [45]. However, the mutation of K48 seems to be not sufficient to affect MxA oligomerization and therefore its antiviral activity. Nevertheless, the expression of SUMO1 and SUMO3 confers stabilization to MxA by enhancing its oligomerization and hence preventing its degradation (Figure 3B) [48]. Thus, SUMO enhances the capacity of MxA to inhibit VSV primary transcription and further induces an intrinsic VSV resistance by stabilizing MxA expression and enhancing its oligomerization. This suggests that MxA proteins may interact with SUMO non-covalently or on other SUMO sites, allowing them to regulate their antiviral activity. Indeed, two putative SIM domains have been identified in the MxA GTPase binding domain, and their mutations reduce MxA antiviral activity (Figure 2) [45].

#### 3.6.2. Phosphorylation Regulates MxB Antiviral Activity

MxB activity is negatively regulated through its phosphorylation [169]. Three sites of phosphorylation have been identified on serines at positions 14, 17 and 18 on the N-terminal domain of MxB (Figure 2). Their dephosphorylation is catalyzed by the myosin light-chain phosphatase (MLCP), reported to promote the antiviral function of MxB. In addition, the phosphomimetic MxB mutant is inactive. Phosphomimetic MxB leads to a reduced localization at the nuclear envelope and a reduced interaction with HIV-1 capsid, thus allowing the virus to recover its ability to enter into the nucleus (Figure 3D). Inversely, IFN treatment, in addition to inducing MxB expression, reduces its phosphorylation promoting MxB antiviral function. Importantly, although we do not know how these mechanisms of regulation operate upon infection, phosphorylation of MxB seems to act as a switch-off for MxB activity.

### 3.7. BST2

The restriction factor bone marrow stromal antigen 2 (BST2, also known as CD317 or tetherin) is a membrane glycoprotein containing a transmembrane domain on its N-terminus, an extracellular coiled-coil domain bearing two N-linked glycosylation sites and a C-terminus modified by a GPI (Figure 1) [59]. BST2 is associated with lipid rafts at the PM and membranes of the trans-Golgi network [59]. Initially identified as the protein that prevents the release of budding HIV-1 particles at the surface of infected cells, BST2 has since been found to block the release of a broad spectrum of enveloped viruses [15,170,171]. BST2 dimerizes via disulfide bonds on its extracellular domain and inserts one of its extremities into the viral membrane during the budding of new particles while the other stays anchored in the host membrane. By its unique conformation, BST2 traps viruses on the cell surface (Figure 1) [172,173].

#### 3.7.1. BST2 Glycosylation Modulates Its Activity

GPI modification of BST2 was shown to be crucial for its antiviral activity. Indeed, this modification allows the trapping of the virus to the PM by BST2, and its depletion impairs the inhibition of virus release such as HIV-1 (delVpu) [172,174], Xenotropic murine leukemia virus-related virus (XMRV) [175], prototypic foamy virus (PFV), bovine foamy virus (BFV) and bovine immunodeficiency virus (BIV) (Figure 3A) [176]. In addition to GPI, BST2 possesses two N-linked glycosylation sites N65 and N92 in the extracellular domain that are both modified, although the residue 65 turns out to be the most important for BST2 antiviral activity (Figure 2) [172,177].

Thus, depletion of both sites and/or inhibition of N-glycosylation greatly impairs BST2 restriction activity against HIV-1 [172,178] and XMRV [175]. This defect of activity is correlated with a decrease in BST2 expression [172,178] and relocalization of BST2 in subcellular compartments [175]. Together, this suggests that glycosylation of at least one N-glycosylation site of BST2 is required for its efficient transport to the PM where it can trap viruses (Figure 3A). The importance of BST2 glycosylation can be extended to other species. Indeed, N-glycosylation sites were reported to be also important for the localization and the antiviral action of feline BST2 [179,180] and equine BST2 [181].

In contrast to these findings, several works showed that N-linked glycosylation was dispensable for the antiviral activity of human BST2 toward HIV-1 [177,182], Lassa and Marburg viruses [171], prototypic foamy virus (PFV) [183] and HBV [182]. Likewise, an unglycosylated mutant of bovine BST2 was still harboring antiviral activity against HIV-1, prototypic foamy virus (PFV) or bovine foamy virus (BFV) [176]. While one study concluded that human N-glycosylated mutant of BST2 does not affect its localization on the cell surface [177], one other revealed that it affects its traffic through the ER membranes and leads to its clustering in CD63-positive vesicles [182].

It is important to note that BST2 transfected in HEK293T consists of two states of glycosylation: principally high-mannose oligosaccharide modification but also complex carbohydrate modifications corresponding to a post-ER form while endogenous BST2 is predominantly in the complex form [177]. Hence, the relevance of incomplete N-glycosylated BST2 expression is important to be determined. Indeed, a complementary approach with chemical inhibitors of the transfer of high-mannose oligosaccharides revealed that complex-type glycosylation is dispensable for both BST2 cell surface expression and antiviral activity toward HIV-1 [178].

Emphasizing the importance of BST2 glycosylation for its antiviral activity, it has been lately shown that SARS-CoV counteracts BST2 restriction by inhibiting its glycosylation [184]. In this work, the authors showed that double mutants of N-linked glycosylation result in the loss of viral restriction against SARS-CoV, without affecting its cell surface localization. ORF7a is a viral transmembrane protein that localizes mainly in the Golgi and is only localized at the cell surface along with BST2 when co-expressed. ORF7a binds directly to unglycosylated BST2 in the Golgi preventing its glycosylation. Then they traffic together to the PM where BST2 is no longer able to operate.

It is commonly agreed that BST2 acts on the PM; however, it has been shown, for example, that, in macrophages, BST2 is localized in virus-containing compartments where BST2 can also trap HIV-1 [185]. This suggests that, depending on the virus studied and the route taken to assemble and bud, it may influence the encounter with BST2. Methods to analyze the level of infection and BST2 expression are various, which could explain the discrepancies in the results.

#### 3.7.2. Viral Antagonisms of BST2 by Its Ubiquitination and Degradation

BST2 was the first described example of a transmembrane protein to be both ubiquitinated and GPI-linked [33]. Two E3 ubiquitin ligases are involved in BST2 constitutive ubiquitination and turnover: NEDD4 and the membrane-associated RING-CH 8 (MARCH8) [34].

Kaposi’s sarcoma-associated herpesvirus (KSHV) and HIV-1 are the two viruses known so far to use the ubiquitin pathway to counteract BST2. Both viruses trigger a downregulation of BST2 from the cell surface. Viral proteins, the viral transmembrane RING-CH E3 ubiquitin ligase K5 of KSHV and Vpu of HIV-1 are responsible for BST2 degradation (Figure 1) [15,33]. Their mechanism of action is independent of the constitutive way involving NEDD4 and MARCH8 [34]. Indeed, K5, which is analog to MARCH8, ubiquitinates BST2 on a single K18 residue located on its cytoplasmic tail during its transfer to the PM (Figure 2) [33,186] while Vpu links BST2 to the beta-transducin repeat-containing proteins (β-TrCPs), a subunit of the Skp1-Cullin1-F-box (SCF) ubiquitin ligase complex, allowing the ubiquitination of BST2 to occur on an STS sequence on its cytoplasmic tail at the PM (Figure 2) [54]. The monoubiquitinated BST2 induced by both K5 and Vpu is recognized by the endosomal sorting complex required for transport I complex (ESCRT-I) [187], triggering its internalization and subsequent degradation along the lysosomal pathway [33,187,188]. Moreover, Vpu is also described to recruit ESCRT-0 that recognizes ubiquitinated proteins for lysosomal degradation (Figure 3E) [189].

## 4. Recent Identified Restriction Factors and Their PTMs

In recent years, an expanding number of studies emerged with screens that have identified other ISGs and antiviral host factors [190,191,192,193]. It is not surprising that these factors are also regulated by several PTMs.

Indeed, a high-throughput imaging-based screen allowed the identification of the mixed-lineage kinase 3 (MLK3) as a restriction factor against Zika virus [190]. MLK3 is a serine/threonine kinase implicated in the Jun N-terminal protein kinase (JNK) pathway that induces cytokine production. Its activation triggered by phosphorylation is induced by Zika virus infection. Recently, the lymphocyte antigen 6 complex locus E (LY6E) was also identified by an ISG screen as an antiviral factor of coronaviruses, including SARS-CoV, SARS-CoV-2 and Middle East respiratory syndrome (MERS)-CoV [191,194]. LY6E inhibits the entry by impeding with spike-protein-mediated membrane fusion [191]. It localizes on the PM thanks to a GPI anchoring probably on lipid rafts where receptors for the virus are also located. Mutating the site of the GPI anchor on the N99 residue abolishes its antiviral activity [194]. Strikingly, for other viruses including flaviviruses, Chikungunya or IAV, LY6E was described to promote infection [195,196], while concerning HIV-1, LY6E can be either disadvantageous or advantageous for the virus, depending on the level of expression of CD4 on target cells [197]. LY6E, as other factors, may be subjected to PTMs modulating positively or negatively its function. However, for the most part of those new factors, more research is required to dissect the role of PTMs in their functions.

Other studies are also evidencing new activities for known antiviral factors. Indeed, in 2015, the death-domain-associated protein 6 (Daxx) was identified as a new restriction factor inhibiting the reverse transcription of HIV-1 and endogenous retroviruses [198]. This protein contains two SIM domains and numerous SUMOylated sites [199,200]. Recently, we reported that Daxx is associated with incoming HIV-1 cores through a SIM-dependent interaction with cyclophilin A (CypA) and capsid (CA) [53]. Interestingly, we found that Daxx, by recruiting TNPO3, TRIM5α and TRIM34 and possibly other proteins onto incoming HIV-1 cores, prevents uncoating and therefore inhibits HIV-1 reverse transcription in a SIM-dependent manner. Thus, this report further suggests that non-covalent interaction with SUMO proteins can be also a critical regulation process in antiviral activity.

Finally, screens for Vpx targets, which was already known to counteract SAMHD1, revealed a new restriction factor also counteracted by ubiquitination-inducing degradation [192,193]. This is the human silencing hub (HUSH) complex composed of three proteins: MPP8, TASOR (Transgene Activation SuppressOR, also named FAM208A) and periphilin that recruits a methyltransferase to mediate repression of transcription [201]. As for SAMHD1, Vpx encoded by HIV-2, but also Vpr from SIV, induces TASOR ubiquitination and degradation, thanks to the DCAF1/CUL4A/B E3 ubiquitin ligase, allowing the transcription of its integrated viral genome. This phenomenon supports again that some viruses can take advantage of the host PTM machinery and puts the antiviral factor modification at the heart of host–pathogen interactions.

## 5. Conclusions

Thereby, in this review we wanted to highlight the fact that regulation of the innate immune system and antiviral defenses are coordinated by a myriad of host enzymes (e.g., E3 ligases, kinases, phosphatases, acetyltransferases) that modify key innate signaling molecules and antiviral factors to fine-tune antiviral responses. These enzymes induce PTMs that act as an on/off switch to modulate protein functions and form a critical part of restriction factor regulation. These factors are highly modified by PTMs, modeling their subcellular localization, stability and activity and regulating protein–protein interaction allowing restriction factors to adapt to viral infections. Nevertheless, evolution between viral proteins and restriction factors are tightly correlated and define the ability of the virus to spread in a particular species. To this end, some viruses evolved by hijacking the PTM machinery to shut them down. Therefore, it will be important to map these factor modifications and to address what determines the specificity of these enzymes toward their target proteins or upon viral infection.

Proteomics studies based on mass spectrometry (LC-MS/MS) applied to certain PTMs make it possible to carry out large-scale studies with great specificity to define a phosphoproteome, acetylome, methylome or SUMOylome. However, given the complexity and dynamics of these interactions, mapping them remains a challenge for years to come. Indeed, it is still unknown whether these regulatory mechanisms are common or differ between different cell types and species. While ubiquitination, phosphorylation and SUMOylation are increasingly associated with antiviral responses and well documented, the roles of other PTMs, such as ISGylation, neddylation, succinylation, carbonylation, glycation, citrullination, nitration and other modifications in intrinsic and innate immunity are still poorly understood. Although signaling networks in which PTMs operate are highly complex and strongly modulated, great progress has been made in recent years. We believe that developing drugs in order to favor PTMs that enhance antiviral factor activity or in order to block viral antagonism and restore efficient restriction is a promising way to fight viral infections and identify more effective therapies.

## Data Availability

Not applicable.
